# Active Ingredients from Foods: Biochemical and Processing Aspects

**DOI:** 10.3390/foods10061240

**Published:** 2021-05-29

**Authors:** Sergio Montserrat-de la Paz

**Affiliations:** Department of Medical Biochemistry, Molecular Biology, and Immunology, School of Medicine, University of Seville, 41012 Seville, Spain; delapaz@us.com

The global food and food technology market is in rapid growth. Food industry’s ability to maintain and strengthen its position and exploit the unique opportunities could be the main point of its growth, especially in the development of high-quality food with relevant sensory health, safety, and sustainability properties as key elements.

Scientific data and epidemiological evidence collected over the last fifty years have shown that nutrition plays a decisive role in human health. The functional role of nutrition is due to specific small molecules with biological activity. These dietary compounds neither act as energy substrates or structural materials for cells, nor as enzymatic cofactors. However, owing to their peculiar bioactivity, human health can be benefited. Bioactive compounds such as polyphenols, carotenoids, vitamins, fatty acids, peptides, organic acids, nucleosides and nucleotides, and phytosterols, mainly in fruits, vegetables, and whole grains, have gained scientific attention due to their preventive role in several chronic diseases.

This Special Issue addresses interdisciplinary research about bioactive ingredients from foods, highlighting their potential for novel applications in the nutraceutical industry. Grape seed is the food studied by Martin et al. because it has demonstrated to present bioactive constituents and is a remarkable winery industry by-product [1]. In this review, the authors speculate that oil obtained from grape seeds is becoming a novel target of food and non-food industries given its various properties. Several sources describe bioactive protein hydrolysates as possible agents in the prevention and treatment of many diseases [2]. Wheat gluten protein hydrolysates could exert potent anti-inflammatory and atheroprotective properties; it was found to reduce the expression of inflammation-related genes and atherosclerosis, and therefore it could be useful in maintaining cardiovascular health, as was demonstrated by Montserrat-de la Paz et al.

Thylakoid membranes isolated from spinach have previously shown capacity to inhibit lipase/co-lipase and prolong satiety in vivo [3]. In this study, spray drying was described as the most suitable drying method yielding a powder with best-maintained functionality after storage [3]. Ostbring et al. results could be useful for quality control of high-quality functional foods with appetite suppressing abilities. In the same line, one of rapeseed’s functional proteins—the amphiphilic protein oleosin—could be used to stabilize emulsions [4]. Rapeseed oleosin recovered from cold-pressed rapeseed press-cake was demonstrated to be a natural emulsifier that exerts anti-oxidation properties [4].

Micronutrients within fruit juice and smoothies are well described to exert benefits on human health, but fewer studies focus on the role of phytochemicals [5]. Bestwick et al. suggested that fruit-based beverage interventions should approach specific mechanisms depending on the fruits from which they are obtained and specifically, the availability of the individual constituents. On the other hand, Cordova et al. studied the effect of drum temperature, drum-rotation frequency, and water-to-pulp ratio in a double-drum drier on the content of glucoraphanin, sulforaphane, ascorbic acid, total phenolic compounds, and antioxidant activity of broccoli pulp through a multilevel factorial design with one replicate [6]. The authors speculate that drum drying represents a very attractive option for the industrialization of broccoli derivatives since it has low investment and operation costs. Finally, a new liquid effluent from alperujo, the main solid by-product from the two-phase olive oil extraction system, has been found; this new liquid, until now had been treated together with the alperujo itself [7]. Fernandez-Prior et al. described that bioactive extracts could be used to add natural components to food, improving their formulation and at the same time, biorefinery would improve the management of the new effluent.

Overall, the research included in this volume covers a wide range of studies, from fundamental research to industry applicability, with respect to health and functional studies in food context. Several of the studies specified the need for more knowledge in their specific spaces and contended that this will lead to better positioned food and food products when looking at world markets. The papers collected in this Special Issue contribute to the growth of this research area. I would like to thank all the authors and the reviewers of the papers published in this Special Issue for their great contributions and effort. I am also grateful to the editorial board members and to the staff of the journal for their kind support during the preparation of this Special Issue.

## Data Availability

No new data were created or analyzed in this study.
